# Glioma‐induced peritumoral hyperexcitability in a pediatric glioma model

**DOI:** 10.14814/phy2.14567

**Published:** 2020-10-07

**Authors:** Lata Chaunsali, Bhanu P. Tewari, Allison Gallucci, Emily G. Thompson, Andrew Savoia, Noah Feld, Susan L. Campbell

**Affiliations:** ^1^ Molecular and Cellular Biology Graduate Program School of Neuroscience Virginia Tech Blacksburg VA USA; ^2^ Fralin Biomedical Research Institute Glial Biology in Health Disease and Cancer Virginia Tech Roanoke VA USA; ^3^ Fralin Biomedical Research Institute Translational Biology, Medicine and Health Virginia Tech Roanoke VA USA; ^4^ Department of Neurobiology Johns Hopkins University Baltimore MD USA; ^5^ Animal and Poultry Sciences Virginia Tech Blacksburg VA USA; ^6^ School of Medicine Virginia Commonwealth University Richmond VA USA

**Keywords:** development, glioma, hyperexcitability, pediatric

## Abstract

Epileptic seizures are among the most common presenting symptom in patients with glioma. The etiology of glioma‐related seizures is complex and not completely understood. Studies using adult glioma patient tissue and adult glioma mouse models, show that neurons adjacent to the tumor mass, peritumoral neurons, are hyperexcitable and contribute to seizures. Although it is established that there are phenotypic and genotypic distinctions in gliomas from adult and pediatric patients, it is unknown whether these established differences in pediatric glioma biology and the microenvironment in which these glioma cells harbor, the developing brain, differentially impacts surrounding neurons. In the present study, we examine the effect of patient‐derived pediatric glioma cells on the function of peritumoral neurons using two pediatric glioma models. Pediatric glioma cells were intracranially injected into the cerebrum of postnatal days 2 and 3 (p2/3) mouse pups for 7 days. Electrophysiological recordings showed that cortical layer 2/3 peritumoral neurons exhibited significant differences in their intrinsic properties compared to those of sham control neurons. Peritumoral neurons fired significantly more action potentials in response to smaller current injection and exhibited a depolarization block in response to higher current injection. The threshold for eliciting an action potential and pharmacologically induced epileptiform activity was lower in peritumoral neurons compared to sham. Our findings suggest that pediatric glioma cells increase excitability in the developing peritumoral neurons by exhibiting early onset of depolarization block, which was not previously observed in adult glioma peritumoral neurons.

## Graphical abstract

The effect of pediatric glioma cells on the function of surrounding neurons during early development is unknown. We intrancranially injected patient‐derived pediatric glioma cells in young pups and examined on the function of peritumoral neurons. Pediatric glioma cells induced changes in the intrinsic properties of periturmoral neurons, lowered the threshold of hyperexcitability and exhibited an early onset of depolarization block.

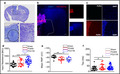

**Key points summary**

- Studies using adult patient tissue and adult glioma models show that the etiology of glioma‐related seizures is complex and not completely understood. No studies have evaluated glioma‐induced changes on neuronal function in early development.
- Using a pediatric glioma model, where pediatric patient‐derived glioma cells were intracranially injected into the cerebrum of postnatal days 2 and 3 (p2/3) pups for 7 days, we examined the effect of glioma on the function of peritumoral neurons.
- The intrinsic properties of pediatric peritumoral neurons were significantly altered. The threshold for eliciting an action potential and pharmacologically induced epileptiform activity was lower in peritumoral neurons compared to sham.
- Peritumoral neurons fired significantly more action potentials in response to small current injection and exhibited depolarization block in response to higher current injection.
- These findings suggest that pediatric glioma cells induce enhanced hyperexcitability in the developing brain by exhibiting depolarization block.

## 1 INTRODUCTION

Gliomas are primary brain tumors derived from cells of the glial (astrocytic and/or oligodendroglial) lineage. Pediatric high‐grade brain tumors are the largest group of pediatric central nervous system (CNS) cancers, the second leading cause of pediatric cancer, and the leading cause of cancer deaths in children (Ward, DeSantis, Robbins, Kohler, & Jemal, 2014; Wilmshurst, Berg, Lagae, Newton, & Cross, 2014). They are also the primary cause of years of potential life lost in children, accounting for 31% of potential life lost (de Blank et al., 2015). Pediatric glioma patients often present with unprovoked seizures leading to tumor‐associated epilepsy, which is often refractory to antiepileptic treatment (Wilmshurst et al., 2014; van Breemen, Wilms, & Vecht, 2007). These uncontrolled seizures are the second most common presenting symptom of supratentorial tumors in children (Gilles et al., 2002; Sánchez Fernández & Loddenkemper, 2017; Stone, Rowell, Jayasekera, Cunningham, & Jacques, 2018; Ullrich et al., 2015). Glioma‐related seizures are closely correlated with the progression and recurrence of gliomas (Di Bonaventura et al., 2017; Liang, 2019). Seizures can dramatically impact a patient's quality of life, cause neurocognitive deterioration and significant morbidity may result from the seizures themselves or medication side effects (Englot, Chang, & Vecht, 2016). Although there are theories regarding the pathophysiology of glioma‐associated epilepsy, the underlying etiology is not fully understood. The tumor type, location, genetics, epigenetics, and microenvironment have been implicated as risk factors (Weller, Stupp, & Wick, 2012). In reference to the latter, there is a stark difference in the tumor microenvironment of pediatric and adult glioma patients, which could affect neuronal changes involved in seizure development. Numerous studies using integrated molecular profiling have established significant differences in specific molecular features that underly pediatric and adult glioma (Brennan et al., 2013; Mackay et al., 2017; Paugh et al., 2010; Sturm et al., 2014; Wu et al., 2014). Furthermore, while data from molecular profiling techniques increasingly show genetic distinctions between adult and pediatric gliomas (Northcott, Pfister, & Jones, 2015; Paugh et al., 2010), fewer studies have examined how these changes impact mechanisms of neuronal hyperexcitability caused by gliomas.

During early development glioma and its associated seizures can impact neurodevelopmental processes and alter neuroplasticity. The developing brain consists of a unique neuronal environment. It is characterized by distinct cortical neuronal network activity that is highly susceptible to synchronized activity (Sanchez & Jensen, 2001). In early development, both intrinsic cellular properties and network architecture contribute to this feature of the immature CNS, which causes seizure incidence to be at its highest in the first year of life, and these seizures are more resistant to antiepileptic drugs (AEDs; Nardou, Ferrari, & Ben‐Ari, 2013). Specific features of the electrophysiological properties of immature neurons involved in these networks are quite different in the early stages of development from those observed in the mature state (Oswald & Reyes, 2008). In particular, the passive neuronal properties of immature neurons such as depolarized resting membrane potential, high input resistance, and low‐threshold calcium currents change significantly in mature neurons, which also receive fewer afferent inputs (Barnett et al., 2014). Together, these distinct features of developing cortical neurons affect their firing properties, which are distinct from mature neurons (McCormick & Prince, 1987). While these differences during early development are required for many important developmental processes, they also render the brain more susceptible to hyperexcitabililty (Barnett et al., 2014). Some of the mechanisms that underlie the activity of immature neurons include electrical coupling between neurons, excitatory actions of GABA, synchronous activation of glutamatergic synapses and intrinsic neuronal bursting (Garaschuk, Linn, Eilers, & Konnerth, 2000; Khalilov, Minlebaev, Mukhtarov, & Khazipov, 2015; Zheng, Lee, & Zhou, 2006). The impact of pediatric glioma cells on the developing brain microenvironment has not been studied.

Using adult human patient samples and adult glioma mouse models, we and others have documented tumor‐associated epilepsy and detailed the mechanisms involved in peritumoral hyperexcitability (Buckingham et al., 2011; Campbell et al., 2015; Hatcher et al., 2020; Pallud et al., 2014; Robert et al., 2015; Tewari et al., 2018). However, there are currently no studies on the effect of pediatric glioma cells on the peritumoral environment in the immature cortex. Knowing the divergent genetics of adult and pediatric gliomas coupled with the differences in the properties of immature and mature neurons, there is a clear need to study the cellular changes that occur in the developing brain environment in response to pediatric glioma cells. Therefore, in the present study, we created two pediatric glioma models in which patient‐derived pediatric glioma cells were intracranially injected into the cerebrum of postnatal day 2 (p2) pups and maintained for 7 days. Using in vitro brain slices from these animals, we examined the effect of pediatric patient‐derived glioma cells on the physiological properties of cortical layer 2/3 peritumoral neurons in the developing cortex. Using whole‐cell patch‐clamp recordings, we found significant changes in the intrinsic properties of peritumoral neurons. In both pediatric glioma models, the resting membrane potential was significantly depolarized compared to sham neurons. In a series of experiments, we discerned that glioma cells in the immature cortex caused peritumoral neurons to be significantly hyperexcitable. In peritumoral neurons the threshold for action potentials (APs) was lower while the firing frequency was higher in response to small current injections. Furthermore, peritumoral immature neurons exhibited spontaneous and evoked epileptiform activity and were more susceptible to chemically induced hyperexcitability. Although immature neurons are known to exhibit depolarization block, pediatric peritumoral neurons displayed enhanced depolarization block in response to smaller current injections. Finally, we determined that the observed changes in pediatric peritumoral neurons’ excitability resulted from the interaction of glioma cells within the pediatric brain environment, as pediatric glioma cells injected into adults did not induce depolarization block. These findings demonstrate that pediatric glioma cells alter cortical neuronal networks to enhance excitability in the immature brain.

## 2 RESULTS

**FIGURE 1:**
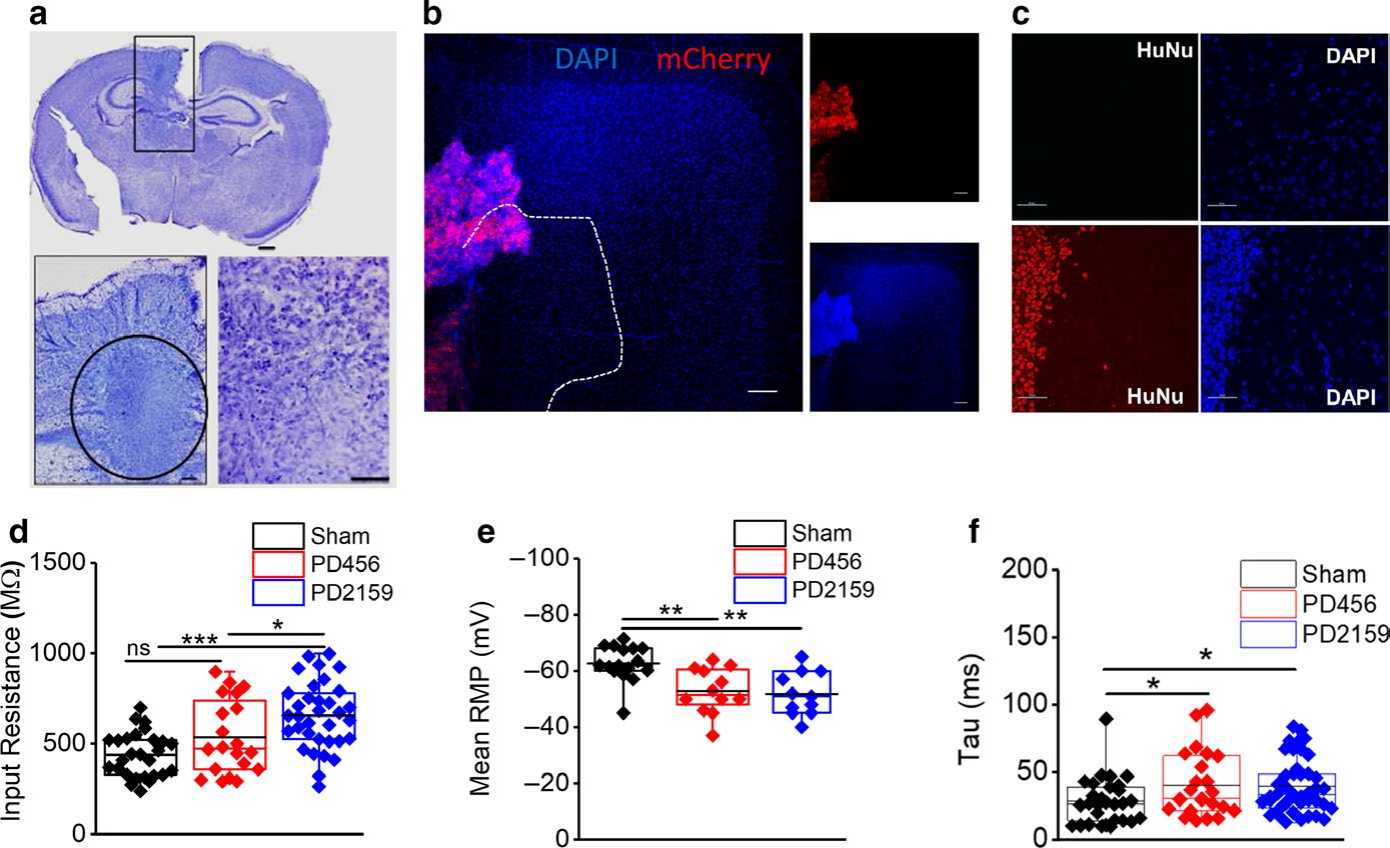
Pediatric glioma cells in the immature cortex. (a) Example of a cresyl violet stained immature cortical brain slice with a mass of PD2159 pediatric glioma cells. Scale bar: top, 500 µm. Bottom, higher magnification of tumor mass. Scale bar: 50 µm. (b) Cortical brain slice showing a mass of PD2159 glioma cells transfected with mCherry (red) traversing the cortical layers, blue is DAPI staining. Scale bar: 100 µm. (c) Identification of HuNu+ (red) PD456 glioma cells in p9 mouse 7 days postimplantation. DAPI staining is in blue. Scale bar: 100 µm. (d) The input resistance of peritumoral neurons in PD21259 is higher and *E*, the RMP is more depolarized compared to sham neurons. (f) Membrane time constant in both types of peritumoral neurons is significantly higher. *p < .05, **p < .01, ***p < .001; one‐way ANOVA with Tukey's multiple comparison post hoc test, asterisks denote significant differences

Using an adult glioma model, we previously reported hyperexcitability in peritumoral neurons leading to spontaneous epileptic seizures (Buckingham et al., 2011). As the brain environment is more susceptible to excitation early in development, we sought to examine functional changes in immature peritumoral neurons using pediatric glioma models. Two glioma models (PD456 and PD2159) were created using glioma cells derived from two pediatric glioma patients. Glioma cells were maintained in the flanks of nude mice and subsequently intracranially injected into the cerebral cortex of p2/p3 mice and allowed to grow for 7 days. By p9 and p10, animals injected with glioma cells showed marked tumor growth in the cortex (Figure 1a). Animals with tumor growth outside of the cortical layers were not included in the study. Whole‐cell patch‐clamp recordings were performed on layer 2/3 pyramidal neurons within ~200–500 µm of the tumor border, termed peritumoral neurons, at p9 and p10 (Figure 1a). Layer 2/3 pyramidal neurons were identified by location and morphology. To better visualize the location and distribution of pediatric glioma cells and demarcation of the peritumoral area in in vitro brain slices, pediatric glioma cells were transfected with mCherry (Figure 1b). Only slices with glioma cells that traversed the cortical layers were used for electrophysiological recordings (Figure 1a,b). Confirmation of the human origin of the tumors was further performed by immunostaining of antibodies against the human‐specific nuclear antigen (HuNu; Figure 1c). In one set of experiments, glioma cells were injected at p2/p3 and recordings were conducted at p17 and p18.

### 2.1 Tumor‐induced changes in the intrinsic properties of pyramidal neurons

In order to address the changes in peritumoral neurons, we first examined the intrinsic membrane properties of pediatric peritumoral and sham neurons by conducting whole‐cell patch‐clamp recordings. The input resistance of peritumoral neurons, measured at the resting membrane potential followed by a small hyperpolarizing step (−20 pA), was significantly higher in PD2159 than sham neurons (sham, 439 ± 23 MΩ, *n* = 27/9; PD2159, 655 ± 31 MΩ, *n* = 34/12; PD456, 535 ± 45 MΩ, *n* = 20/7, *p* < .01 (*p* = .0000172755 sham vs. PD2159; *p* = .04128 PD2159 vs. PD456), one‐way ANOVA with Tukey's multiple comparison post hoc test; Figure 1d). The resting membrane potential (RMP) was significantly depolarized (sham, −62 ± 1 mV, *n* = 16/7; PD2159, −51 ± 2 mV, *n* = 11/5; PD456, −52 ± 2 mV, *n* = 12/5, *p* < .01 [*p* = .00324], sham vs. pd456; *p* = .00155, sham vs. PD2159) one‐way ANOVA with Tukey's multiple comparison post hoc test; Figure 1e), while the membrane time constant (*τ*_m_) was significantly increased in both groups of peritumoral neurons compared to sham (sham, 28 ± 3.2 ms, *n* = 29/9; PD2159, 39 ± 3.1 ms, *n* = 23/9; PD456, 40 ± 5 ms, *n* = 42/13, *p* < .05, one‐way ANOVA, Fisher's LSD multiple comparison post hoc test; Figure 1f). These findings confirm that the presence of glioma cells significantly alters the intrinsic properties of pediatric peritumoral neurons.

### 2.2 Pediatric peritumoral neurons exhibit more negative AP threshold

**FIGURE 2:**
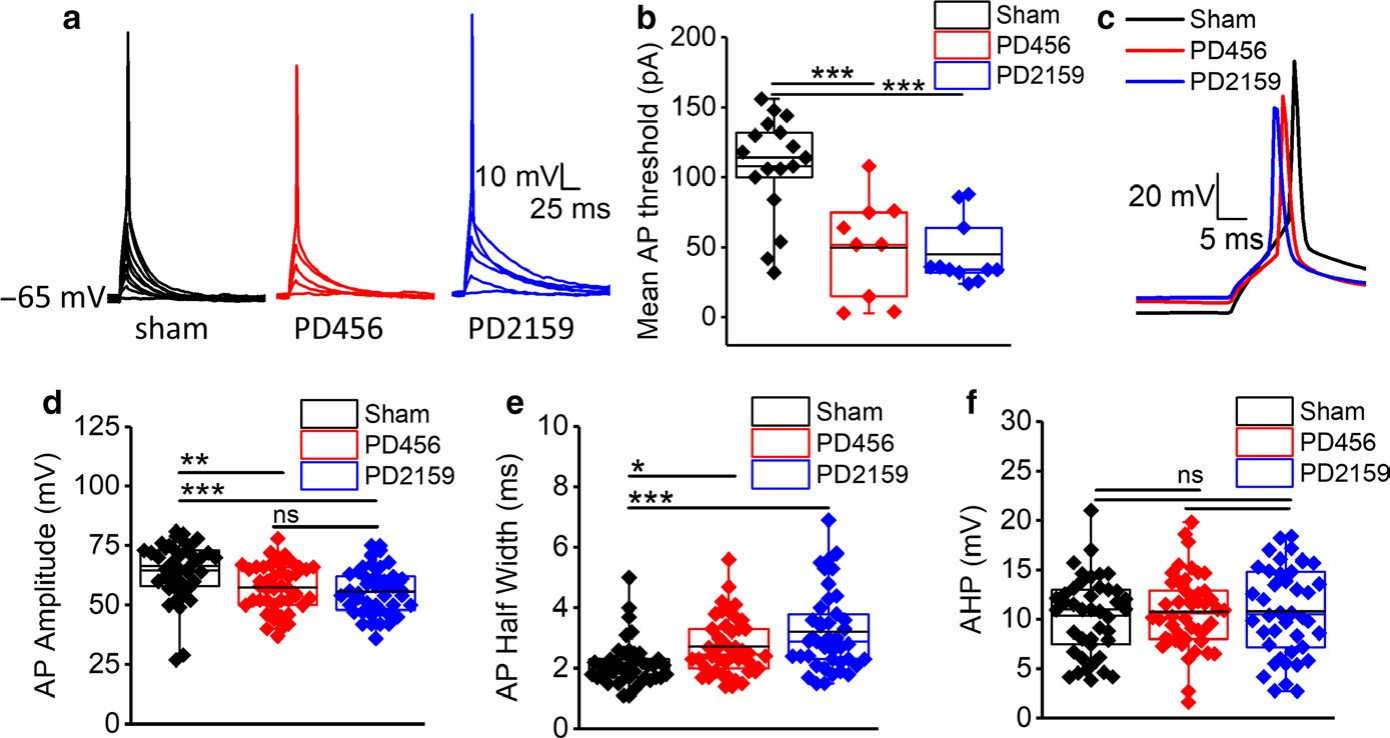
Pediatric peritumoral neurons have decreased action potential threshold. (a) Current‐clamp recordings depicting the depolarizing current pulse required to evoke an action potential in sham and peritumoral (PD456, PD2159) neurons. (b) Quantification shows less current is required to trigger an action potential in peritumoral neurons compared to sham. (c) Examples of individual traces of action potentials from peritumoral and sham neurons. (d) Quantification of action potential amplitude shows a decrease in peritumoral neurons. (e) The AP half‐width of peritumoral neurons was greater than sham neurons, while there were no changes in their (f) after hyperpolarization amplitude. *p < .05, **p < .01, ***p < .001; one‐way ANOVA with Tukey's multiple comparison post hoc test, asterisks denote significant differences

To better understand how changes in the intrinsic properties of peritumoral neurons influence the firing properties and consequently neuronal excitability in peritumoral brain, we measured the threshold of AP generation, AP amplitude, and the half‐width of APs in peritumoral neurons compared to sham neurons. The threshold of APs was determined by injecting current in small increments (2 pA) for 10 ms duration. Peritumoral neurons required significantly less current injection to elicit the first AP compared to sham neurons. Therefore, the AP threshold in peritumoral neurons was significantly more negative than that of sham neurons (sham, 107 ± 8 pA, *n* = 17/7; PD2159, 44 ± 6 pA, *n* = 11/5; PD456, 49 ± 12 pA, *n* = 9/6, *p* < .001 (*p* = .000432539, sham vs. pd456; *p* = .000060972, sham vs. pd2159) one‐way ANOVA, Tukey's multiple comparison post hoc test; Figure 2a,b). Action potential amplitudes were measured from the onset to the peak. Figure 2c illustrates that pediatric peritumoral neurons displayed smaller AP amplitudes than sham neurons (sham, 64 ± 1 mV, *n* = 42/12; PD2159, 55 ± 1 mV, *n* = 42/13; PD456, 57 ± 1 mV, *n* = 46/13, *p* < .01 (*p* = .000606614, sham vs. PD2159; *p* = .00532, sham vs. PD456) one‐way ANOVA with Tukey's multiple comparison post hoc test; Figure 2c,d). In addition to the decrease in amplitudes, AP kinetics in peritumoral neurons also changed. The AP half‐width was greater in peritumoral neurons compared to sham (sham, 2.1 ± 0.1 ms, *n* = 42/12; PD2159, 3.2 ± 0.2 ms, *n* = 42/13; PD456, 2.7 ± 0.1 ms, *n* = 46/13, *p* < .01 (*p* = .00002, sham vs. PD2159; *p* = .03499, sham vs. PD456) one‐way ANOVA with Tukey's multiple comparison post hoc test; Figure 2e). There were no significant differences in the after‐hyperpolarization amplitudes (sham, 10.3 ± 0.6 mV, *n* = 42/12; PD2159, 10.7 ± 0.6 mV, *n* = 42/13; PD456, 10.7 ± 0.5 mV, *n* = 46/13, *p* > .05 (*p* = .88284, sham vs. PD2159; *p* = .90256, sham vs. PD456; *p* = .99834, PD2159 vs. PD456) one‐way ANOVA with Tukey's multiple comparison post hoc test; Figure 2f). Together these findings suggest that glioma cells induce changes in the surrounding neurons to make them more susceptible to depolarize and fire APs. This shift in AP threshold is likely to elicit dramatic hyperexcitability in the immature brain, which is already prone to excitation.

### 2.3 Peritumoral neurons exhibit enhanced spontaneous AP firing

**FIGURE 3:**
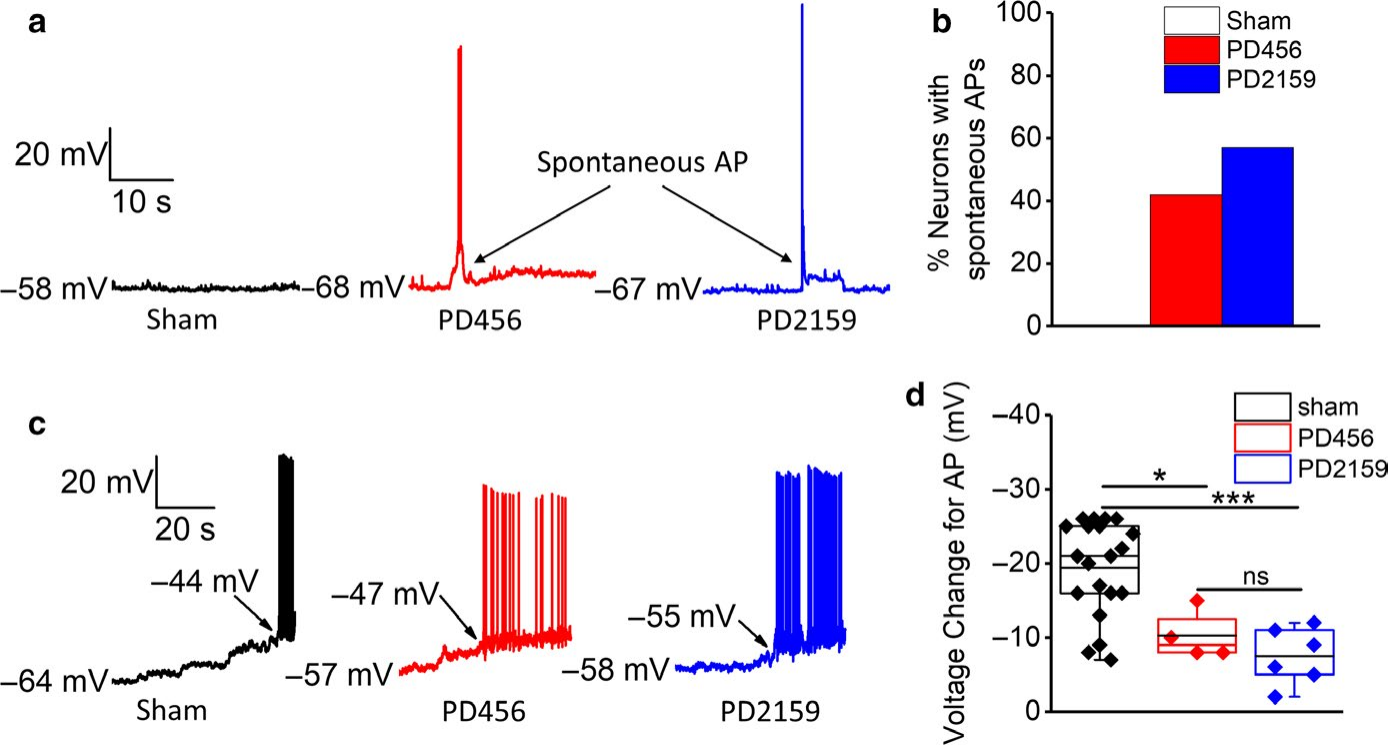
Peritumoral neurons fire spontaneous action potentials. (a) Representative traces of current‐clamp recordings of spontaneous voltage fluxes showing peritumoral neurons firing spontaneous action potentials. (b) quantification of the percent of cells that fire spontaneous action potentials, showing that peritumoral neurons from both pediatric glioma models exhibit spontaneous action potentials while sham neurons do not. (c) representative traces of current‐clamp recordings depicting the magnitude of depolarizing voltage change required for cells to fire action potentials, *left* sham, *middle* PD456 and *right* PD2159. (d) quantification of the voltage change required for cells to fire action potentials. *p < .05, ***p < .001; one‐way ANOVA with Tukey's multiple comparison post hoc test, asterisks indicate significant differences

The data above suggest a basal increase in the excitability of peritumoral neurons that could translate into a gradual built‐up of excitatory drive to generate spontaneous APs and result in hyperexcitability. To examine this possibility, we conducted current‐clamp recordings of spontaneous voltage fluxes in peritumoral and sham neurons to determine if peritumoral neurons fire spontaneous APs. We found that sham neurons do not exhibit spontaneous APs (even at a more depolarized resting membrane potential), while most peritumoral neurons fire spontaneous APs at depolarized RMPs; some peritumoral neurons were observed to fire APs even at hyperpolarized RMPs (Figure 3a). Compared to 0% of sham, 57% (8/14) of PD2159‐ and 42% (12/28) of PD456‐implanted peritumoral neurons exhibited spontaneous APs (Figure 3b). Next, we measured the voltage change required for cells to fire APs. We manually depolarized the cells from their RMPs until they fired their first AP and then determined the mean change in voltage. The voltage change required for sham neurons to fire APs is significantly higher than that required for peritumoral neurons (sham, −19 ± 1 mV, *n* = 20/6; PD2159, −7 ± 1 mV, *n* = 6/4; PD456, −10 ± 1, *n* = 4/4. *p* < .05 (*p* = .00033916, sham vs. PD2159; *p* = .01768, sham vs. PD456) one‐way ANOVA with Tukey's multiple comparison post hoc test, Figure 3c,d).

### 2.4 Pediatric glioma cells induce increased AP firing in peritumoral neurons and exhibit enhanced depolarization block

**FIGURE 4:**
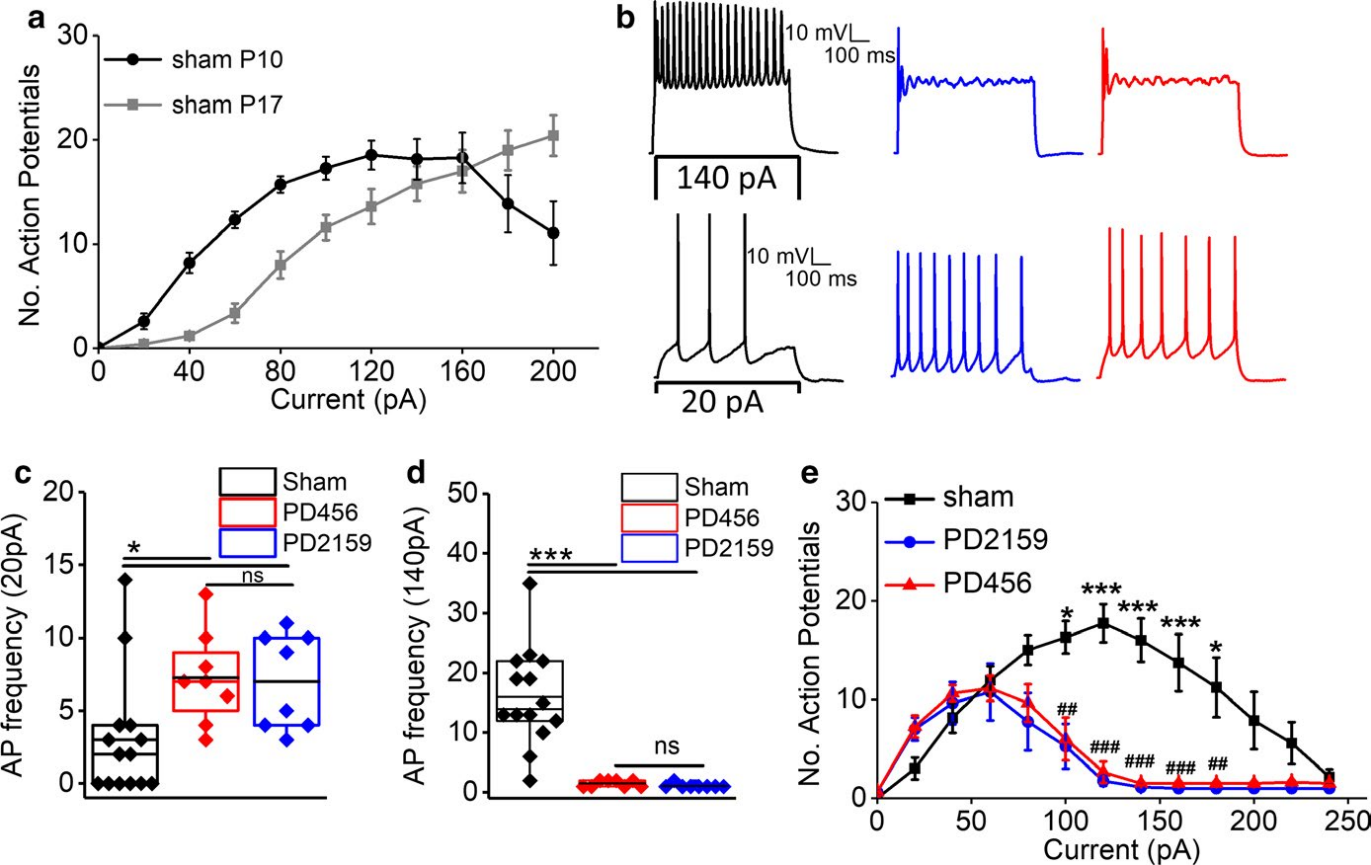
Pediatric glioma cells induce enhanced depolarization block in peritumoral neurons. (a) Plot of the average number of action potentials versus current input of p9/p10 and p17/p18 sham neurons. The younger p9/p10 neurons exhibit depolarization block while the older p17/p18 neurons show an increasing number of action potentials in response to increasing current injections. (b) Representative traces of 40 and 140 pA current injections of sham, PD456 and PD2159 peritumoral neurons. (c) Quantification of the number of action potentials at 20 pA (*p < .05, one‐way ANOVA with Fisher's LSD multiple comparison post hoc test), and (d) at 140 pA (***p < .001, one‐way ANOVA with Tukey's multiple comparison post hoc test); asterisks refer to significant differences, ns means no significant difference. (e) Input/output curve showing an initial leftward shift in the peritumoral neurons’ curve at lower amplitude current injections and a complete depolarization block in peritumoral neurons in response to higher current injections (*p < .05, ***p < .001, two‐way ANOVA with Tukey's multiple comparison post hoc test for sham and PD456; ^#^ *p* < .05, ^##^ *p* < .01, ^###^ *p* < .001, two‐way ANOVA with Tukey's multiple comparison post hoc test for sham and PD2159)

With chronic excitation, depolarization block occurs causing an attenuation of AP amplitude and failure of AP generation (Kim & Nykamp, 2017). Studies have shown that depolarization block occurs in neurons from young animals during early development but not in the mature neurons of older animals after a developmental switch occurs (Oswald & Reyes, 2008). Therefore, we first evaluated the ability of neurons from young animals at p9/10 and neurons from older p17 animals to maintain AP firing by increasing the amplitude of depolarizing current injections (0 to +200 pA) for 500 ms. In p9/p10 neurons from sham animals, increasing the current injection (0 to 120 pA) caused an increase in the number of APs; however, further increases in current injection (140–200 pA) caused a decrease in the number of APs with broadening of successive spikes and eventual failure of AP generation leading to depolarization block (Figure 4a). By comparison, increasing current injections in p17 sham neurons caused a uniform increase in the number of APs without decrement at higher current injections (Figure 4a).

Upon establishing the occurrence of depolarization block in p9/10 pediatric sham neurons, we cautiously selected the minimum injected current that: (a) minimally depolarizes neurons to generate APs (20pA) and, (b) generates the highest spike frequency without any depolarization block (140pA). We then used these currents to stimulate p9/10 sham neurons and compared their responses with peritumoral neurons. Surprisingly, peritumoral neurons exhibited completely opposite features in response to these specific currents. We observed an enhanced excitability at the lower threshold current of 20 pA in both peritumoral neuron groups compared to a significantly lower excitability in age‐matched sham controls (sham, 3 ± 1.1 APs, *n* = 14/5; PD2159, 7 ± 1 APs, *n* = 8/4, PD456, 7.2 ± 1.1, *n* = 8/5; *p* < .05 (*p* = .01596, sham vs. PD456; *p* = .02252, sham vs. PD2159) one‐way ANOVA with Fisher's LSD multiple comparison post hoc test; Figure 4b top traces, c). In addition, injecting the minimum current that generated the highest spike frequency without any depolarization block in p9/10 shams (140 pA) caused depolarization block in both groups of peritumoral neurons (sham, 16 ± 3.1 APs, *n* = 14/5; PD2159, 1.12 ± 0.1 APs, *n* = 8/4, PD456, 1.5 ± 0.5, *n* = 8/5; *p* < .001 [*p* = .000012432, sham vs. PD456; *p* = .00000839411, sham vs. PD2159] one‐way ANOVA with Tukey's multiple comparison post hoc test; Figure 4b bottom traces, d).

To precisely measure the differences in excitability and depolarization block of peritumoral neurons compared to shams, we obtained complete input–output curves over a series of current injections (+20 pA to +240 pA), and the number of APs fired by peritumoral neurons declined while sham neurons exhibited an increase (*p* < .001, two‐way ANOVA with Tukey's multiple comparison post hoc test; Figure 4e). With increasing current injections, the APs in peritumoral neurons started to attenuate sooner than in sham neurons (sham, 160 pA, *n* = 14/5; PD2159, 60 pA, *n* = 8/4; PD456, 60 pA, *n* = 8/4, *p* < .01 two‐way ANOVA with Tukey's multiple comparison post hoc test; Figure 4e). The changes in AP attenuation and depolarization block were observed in peritumoral neurons from both pediatric glioma models, suggesting that glioma cell interactions in the developing brain microenvironment cause significant changes in neurons to prevent AP firing.

### 2.5 Enhanced spontaneous and evoked epileptiform activity in pediatric peritumoral neurons

**FIGURE 5:**
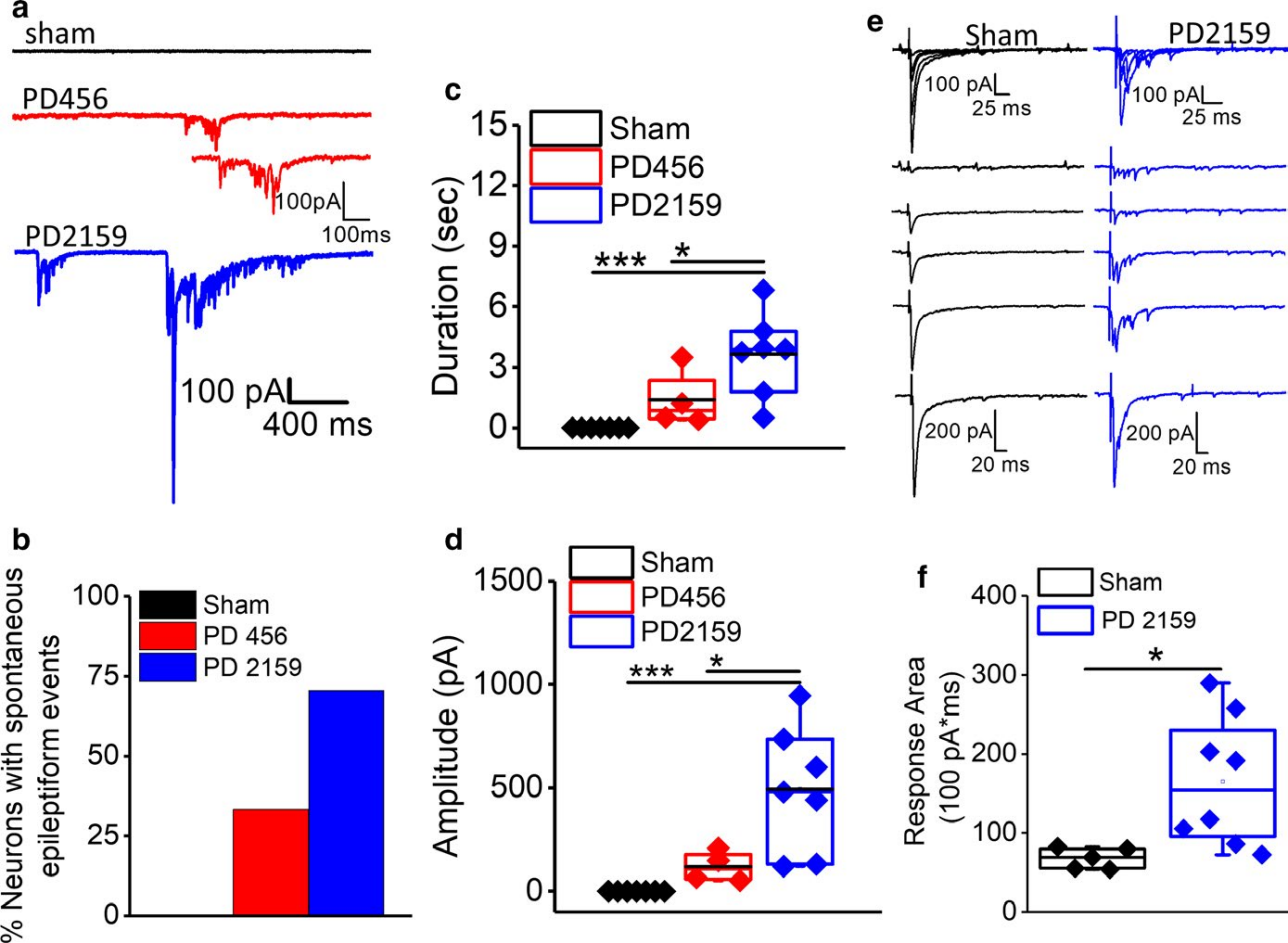
Peritumoral neurons exhibit spontaneously occurring epileptiform activity and evoked epileptiform activity. (a) Representative current traces from sham, PD456 and PD2159 peritumoral neurons showing spontaneous epileptiform discharges. (b) Percentage of neurons showing spontaneous epileptiform events in sham, PD456 and PD2159 peritumoral area. (c) Box and whisker plot showing the duration of spontaneous epileptiform discharges in glioma groups and sham animals (*p < .05, ***p < .001, one‐way ANOVA, Fisher's post hoc test). (d) Box and whisker plot showing the amplitude of spontaneous epileptiform discharges in glioma and sham groups (*p < .05, ***p < .001, one‐way ANOVA, Tukey's post hoc test). (e) Representative current traces from sham and PD2159 peritumoral neurons showing field stimuli evoked postsynaptic currents. (f) Box and whisker plots showing a significantly higher response area of evoked postsynaptic currents in PD2159 neurons than sham (*p < .05, two‐tailed unpaired *t* test)

These results show that pediatric peritumoral neurons are more depolarized and excitable, which could culminate in generation of spontaneously occurring epileptiform activity in the immature cortex. Therefore, we surveyed whether peritumoral neurons exhibited spontaneously occurring epileptiform events by recording spontaneous postsynaptic currents (sPSCs) for 10 min. We found that peritumoral neurons exhibited spontaneously occurring epileptiform activity, which did not occur in sham neurons (sham, 0/15, 0%; PD2159, 12/17, 70.5%, PD456, 4/12, 33.3%; Figure 5a,b). The epileptiform events in PD2159 were longer in duration (sham, 0.0 ± 0.0 s, *n* = 7/4; PD2159, 3.64 ± 0.77 s, *n* = 7/4; *p* < .001 [*p* = .000269013], one‐way ANOVA, Fisher's post hoc test) compared to PD456 (PD456, 1.40 ± 0.71 s, *n* = 4/3; *p* < .05 [*p* = .0254], one‐way ANOVA, Fisher's post hoc test, Figure 5c). The amplitude of epileptiform events was also significantly higher in the PD2159 group compared to sham (sham, 0.0 ± 0.0 pA, *n* = 7/4; PD2159, 493.33 ± 114.13 pA, *n* = 7/4; *p* < .001 [*p* = .000693343], one‐way ANOVA, Fisher's post hoc test) and PD456 (PD456, 118.60 ± 36.78 pA, *n* = 4/3; *p* < .05 [*p* = .01945], one‐way ANOVA, Fisher's post hoc test Figure 5d).

Next, we used peritumoral neurons from animals with PD2159 glioma cells to test whether stimulation could evoke epileptiform events. Evoked postsynaptic currents (ePSCs) were elicited by intracortical stimulation with increasing stimulation intensity under control conditions. Evoked currents from sham neurons displayed a uniform increase in response amplitude with increased stimulation intensity (Figure 5e). However, evoked currents in peritumoral neurons displayed abnormal polysynaptic events with a significantly longer response area (sham, 6,811.9 ± 596.9 (ms*pA), *n* = 5/3; PD2159, 16,532.1 ± 2,891.1 (ms*pA), *n* = 8/4; *p* < .05 (*p* = .024) two‐tailed unpaired *t* test, Figure 5e,f).

**FIGURE 6:**
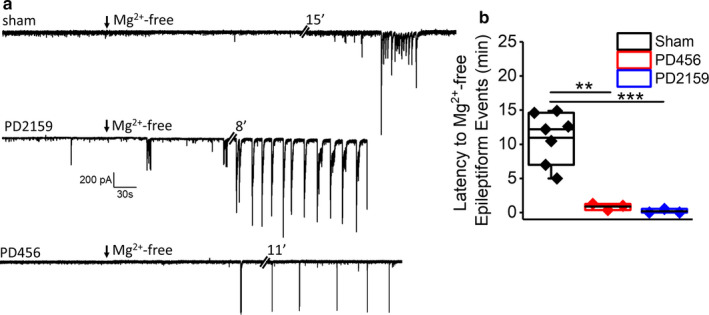
Shorter latency of evoked epileptiform discharges in peritumoral neurons on Mg^2+^‐ free ACSF perfusion. (a) Representative current traces from sham, PD2159 and PD456 peritumoral neurons showing onset of epileptiform discharges at different time points after Mg^2+^‐ free ACSF perfusion. (b) Box and whisker plot showing significantly shorter latency of epileptiform discharge onset in PD456 and PD2159 peritumoral neurons after Mg^2+^‐free ACSF perfusion (**p < .01, ***p < .001, one‐way ANOVA, Tukey's post hoc test, asterisks refer to significant differences)

To further detail pediatric peritumoral hyperexcitability, we also assessed the latency to chemically induced hyperexcitability. Following baseline recordings, we created a proconvulsive environment by removing extracellular Mg^2+^ from the ACSF, which induced epileptiform activity in all neurons. Calculation of the latency to the first epileptiform event revealed a significantly shorter latency in peritumoral neurons compared to sham neurons (sham, 10.9 ± 1.4 min, *n* = 7/4; PD2159, 0.17 ± 0.17 min, *n* = 3/3; PD456, 0.8 ± 0.2, *n* = 3/3; *p* < .01 [*p* = .00084177, PD2159 vs. sham; *p* = .00138, PD456 vs. sham] one‐way ANOVA with Tukey's multiple comparison post hoc test, Figure 6a,b). The observed enhancement in spontaneous and evoked epileptiform activity indicates that pediatric glioma cells induce hyperexcitability in peritumoral neurons.

**FIGURE 7:**
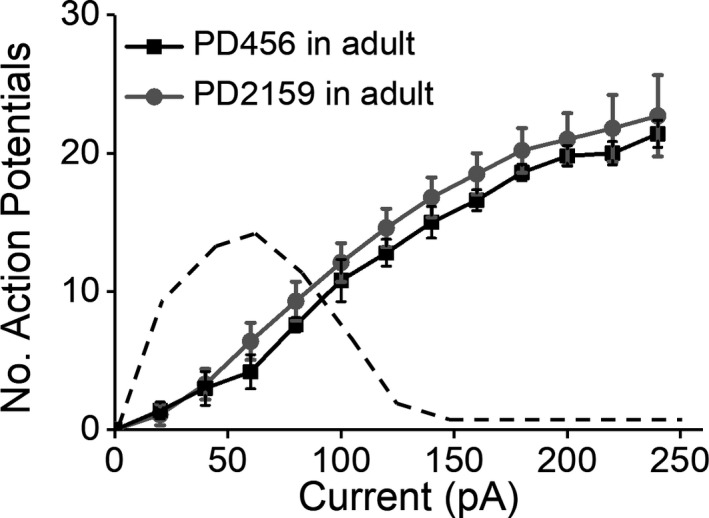
Pediatric glioma does not induce depolarization block in peritumoral neurons in adult brain. Input–output curve of peritumoral adult neurons that were injected with PD456 and PD2159 glioma cells showing the absence of depolarization block and no significant differences in spike frequency (*p* > .05, two‐way ANOVA, Tukey's post hoc test). Dash line indicates depolarization observed in pediatric glioma cells

The observed changes in the properties of peritumoral neurons in these pediatric glioma models could result from inherent properties of the pediatric glioma cells themselves, such that they exert changes in any peritumoral milieu (pediatric or adult) that glioma cells from adult patients do not. Alternatively, the changes in pediatric peritumoral neurons could result from the interactions of pediatric glioma cells specifically within the immature brain environment. To determine if the properties of pediatric glioma cells were sufficient to drive adult peritumoral neurons to exhibit depolarization block and hyperexcitability, we injected pediatric glioma cells into the cerebrum of adult mice and assessed the firing properties of peritumoral neurons. The input–output curve in Figure 7 shows that when pediatric glioma cells (PD2159 or PD456) were injected into adult cortex, peritumoral neurons displayed increasing numbers of APs in response to higher current injections but neither exhibited depolarization block (Figure 7, PD2159, *n* = 10/5, PD456, *n* = 5/4, *p* > .05; two‐way ANOVA, Tukey's multiple comparisons post hoc test). Taken together, these results suggest that the observed increase in neuronal hyperexcitability results in part from the interaction of pediatric glioma cells with the developing brain environment.

## 3 DISCUSSION

In the present study, we created two pediatric glioma models to examine the impact of pediatric glioma cells on the function of neurons in the immature cortex. To our knowledge, this is the first study to examine functional changes in peritumoral neurons in a pediatric glioma model. In our model, intracranial injection of pediatric glioma cells into immature cortex at p2/3 results in rapid tumor growth in the developing cortex within 7 days. Using electrophysiological recordings, we report four key findings. First, the intrinsic properties of pediatric peritumoral neurons are significantly altered. Second, changes in peritumoral neuron properties are accompanied by increased AP firing in response to low amplitude current injections. Third, although small current injections in peritumoral neurons cause more APs, injection of larger currents leads to depolarization block sooner than in sham neurons. Finally, peritumoral neurons exhibit spontaneously occurring epileptiform events and are more susceptible to chemically induced hyperexcitability. These findings suggest that, in the immature cortex, pediatric peritumoral neurons exhibit enhanced vulnerability to hyperexcitability. These studies also confirmed that intracranial injection of pediatric glioma cells into young pups results in tumor growth which can be utilized as a reproducible pediatric glioma model.

### 3.1 Glioma and epilepsy

Although gliomas in children are relatively rare compared to adults, when they are located in the cortex both age groups present with seizures (Thom, Blumcke, & Aronica, 2012; van Breemen et al., 2007). Due to the lower occurrence in children, it is not surprising that most research on glioma‐associated epilepsy focuses on adult glioma models. The biology, genetics, and epigenetics of glioma cells in adults and children are also different, and could influence seizure susceptibility (Liang, 2019). The general assumption that adult and pediatric patients have similar mechanisms of seizure development completely ignores the vast majority of studies showing fundamental differences in the physiochemical attributes of neurons in adult and immature brains (Holmes, 1997). In addition to the physiological differences, the pathophysiology of epilepsy in the two age groups is also distinct, including differences in their seizure susceptibility, seizure characteristics, and responses to AEDs (Holmes, 1997). One contributing factor is the paucity of adequate experimental models to specifically investigate how pediatric glioma cells affect immature neuronal networks. Neurons in the immature brain exhibit a wide range of developmental differences compared to the adult brain, including a higher level of synaptogenesis and neuronal plasticity, high synaptic density, elevated expression of glutamate receptors, and differences in the expression and molecular composition of neurotransmitter receptors and transporters, voltage gated ion channels and mechanisms of ionic homeostasis (Sanchez & Jensen, 2001). These developmental processes may be differentially affected by glioma cells in the immature brain.

### 3.2 Pediatric peritumoral neurons’ intrinsic properties

Before investigating the changes in peritumoral neurons, we first had to visualize tumor cells following intracranial injection. Unlike adult tumor‐bearing slices, tumor cells are not easily detected by eye in 300‐µm‐cortical slices from young p9/p10 mice. In these slices, tumor cells were detected by visualization under a 40X objective. This difference is caused by the lack of myelination in p9/10 brain tissue, which is similar in appearance to tumor cells, whereas the adult brain is highly myelinated. Therefore, to better delineate the tumor border, we transfected tumor cells with mCherry. To ensure that transfection did not disturb the genetic features of glioma cells, we evaluated and compared the expression of several genes in transfected and nontransfected glioma cells and found no differences (data not shown).

In early development, cortical neurons exhibit unique physiological properties including a higher input resistance, membrane time constant, and intracellular chloride concentration, which drives an excitatory GABA response (Ben‐Ari, 2002). They also exhibit a smaller rheobase and lower RMP (Oswald & Reyes, 2008). Although these features are important for many developmental processes that are required for brain maturation, including neuronal growth, synapse formation and network wiring, they also predispose the brain to be more hyperexcitable (Jensen, 1999; Rakhade & Jensen, 2009; Sanchez & Jensen, 2001). During normal development, these properties change over time, leading to a tightly regulated balance of excitation and inhibition in the mature brain. The presence of glioma cells can certainly change this balance, and the region adjacent to the tumor border is considered to be the crucial area involved in generating hyperexcitability in both adult patient tissue and adult glioma models (Buckingham et al., 2011; Paugh et al., 2010; Robert et al., 2015). Our results show that the intrinsic properties of layer 2/3 peritumoral pyramidal cells from p9/10 mice, including input resistance and membrane time constant, are significantly increased. Changes in their intrinsic properties could have profound effects not only on the cells’ intrinsic excitability, but also on the aforementioned developmental processes. It is unknown how enhanced and/or prolonged excitability induced by glioma cells in the immature brain impacts these developmental processes.

### 3.3 Enhanced firing and depolarization block in immature neurons

Our results show that pediatric peritumoral neurons exhibit depolarization block in response to smaller current injections than sham neurons. Depolarization block is a physiological state in which the neuronal membrane is depolarized, but action potentials cannot be triggered. This is observed in in vitro brain slices when neurons cease firing when a stimulus is too strong (Bianchi et al., 2012). To understand the mechanism involved in the generation of depolarization block, several models have been used to investigate different parameters that could affect this phenomenon. In immature neurons, depolarization block has been reported in the auditory cortex of p10/p11 mice; as development continues, the response transitions to increased AP firing at higher stimulation intensities by p18 (Oswald & Reyes, 2008). These developmental changes in neurons in the auditory cortex play an important role in the function of auditory pathways (Seidl & Grothe, 2005) and are consistent with our observation that depolarization block did not occur in our p17/p18 sham neurons. There was a significant rightward shift in the I/V curve of sham neurons at p17/p18 compared with younger p9/10 sham neurons. While the exact mechanism for depolarization block is unknown, several mechanisms have been proposed including inactivation of voltage‐dependent Na+ channels and alteration of extracellular potassium concentration (Bianchi et al., 2012; Kim & Nykamp, 2017; Tucker, Huertas, Horn, Canavier, & Levitan, 2012). Depolarization block is reported to be preceded by attenuation of AP amplitude and broadening of each successive spike, leading to the eventual failure of AP production (Blythe, Wokosin, Atherton, & Bevan, 2009; Richards, Shiroyama, & Kitai, 1997). In another model, cortical neurons also exhibited shortened AP heights and depolarization block at higher current amplitudes. Furthermore, increasing the temperature from 34 to 37°C caused depolarization block to occur at even lower current amplitudes, due to faster Na+ inactivation kinetics (Aberra, Peterchev, & Grill, 2018). Our data are consistent with those findings, as the amplitude of pediatric peritumoral neurons’ APs decreased while the half‐width increased prior to depolarization block.

### 3.4 Tumor microenvironment

The tumor microenvironment plays an important role in peritumoral hyperexcitability and glioma progression. Previous findings described the interplay between gliomas and neurons showing that gliomas can integrate into electrical networks and that depolarization of the glioma cells promotes glioma progression (Venkataramani et al., 2019). To facilitate this, glioma cells express synaptic genes including glutamate receptor genes and postsynaptic structural genes that resemble oligodendroglial precursor cells (Venkatesh et al., 2019). It is unknown if similar glioma‐induced genetic changes occur in the developing brain and contribute to neuronal hyperexcitability. Furthermore, electron microscopy studies also revealed the presence of glioma cells on the postsynaptic side of synaptic structures (Venkataramani et al., 2019; Venkatesh et al., 2019). In these studies, glioma cells engrafted in the CA1 region of the hippocampus was also shown to respond to stimulation with fast inward currents (Venkatesh et al., 2019). Our recordings were conducted in the cortex, however, it is possible that interaction of synaptic glioma within the developing brain could contribute to the hyperexcitable peritumoral neurons reported in our study. In another study, glioma that was generated via in utero deletion of key tumor suppressor genes revealed reproducible neuronal hyperexcitability and behavioral seizures (Hatcher et al., 2020). Together, these data support an intimate relationship between glioma cells and peritumoral neurons which impacts neuronal network. While peritumoral neurons in both the PD456 and PD2159 groups exhibited enhanced excitability and spontaneous epileptiform activity, the enhanced excitability was more pronounced in the PD2159 group. Among other possibilities, this could result from genetic differences in the glioma cells.

In this study, we gained insights into the effects of pediatric glioma cells on the activity of peritumoral neurons in the context of the immature cortex. However, characterizing the exact mechanisms underpinning the observed excitability in peritumoral neurons is beyond the scope of this work. In adult glioma models, glutamate released from tumor cells, a decrease in inhibitory neurons, excitatory actions of GABA and alterations in perineuronal nets contribute to peritumoral hyperexcitability (Campbell et al., 2015; Robert et al., 2015; Tewari et al., 2018). These mechanisms cannot be extrapolated to the immature brain as both clinical and animal studies suggest a unique relationship between brain maturation and epilepsy (Jensen, 1999; Sanchez & Jensen, 2001). Therefore, further studies are required to mechanistically probe the relationship between glioma cells in the immature brain and the neuronal hyperexcitability that causes seizures. Since most of our previous studies assessed changes in the peritumoral environment, it is unknown if the electrophysiological properties of pediatric glioma cells differ from adult glioma cells. Although these findings underscore the importance of glioma‐neuronal networks in the function of peritumoral neurons, these networks may be different in the developing brain.

Our data suggest that the presence of glioma alters the biophysical properties of peritumoral neurons and shifts the excitatory–inhibitory balance toward excitation, leading to the generation of spontaneous epileptiform activity and an increased susceptibility to hyperexcitability. The immature state of the brain exacerbates this effect. The intrinsic differences in pediatric and adult brains suggest a unique interplay between gliomas and the microenvironment of the pediatric brain such that the already seizure prone immature brain is even more easily driven to exhibit spontaneous seizure activity. Further studies are needed to determine precisely how the presence of glioma alters the biophysical properties of peritumoral neurons to increase hyperexcitability.

### 3.5 Ethical approval

All animal procedures were approved and performed in accordance with the ethical guidelines set by Virginia Tech Institutional Animal Care and Use Committee (IACUC). Animals were maintained in a specific pathogen‐free barrier facility in 12 hr light/dark cycles with free access to food and water. Male and female C.B.17 scid mice aged p2–p40 were used for intracranial tumor implantation. Female athymic nude mice aged 6–8 weeks were used for flank injections, maintenance and propagation of GBM xenografts. All mice were maintained under standard laboratory conditions with 12:12 hr light/dark cycle and ad libitum access to water and food.

The pediatric PD456/D456MG and PD2159/D2159MG xenografts were acquired from Dr. Darell Bigner at Duke University Medical Center and all procedures for obtaining these biopsies for tissue were approved by the Duke University Institutional Review Board (#Pro00007434). Informed and written consents were included in the IRB approvals and obtained from all patients involved in these tissue collection procedures. PD456 and PD2159 were previously established from human biopsies, 4‐ and 5‐year‐old, respectively.

### 3.6 Patient‐derived xenograft tumors

Patient‐derived primary glioma tissue was maintained by serial passage in nude mice flanks as described previously (van Breemen, Wilms, & Vecht, 2007). Briefly, tumors were harvested 14–18 days postinjection and glioma cells were mechanically and enzymatically dissociated. Cells were passed through a 40‐µm filter and maintained as “gliospheres” in Dulbeccoʼs modified Eagle medium/nutrient mixture F‐12 (DMEM/F‐12), supplemented with 10‐mg/ml fibroblast growth factor (FGF), 10‐mg/ml epidermal growth factor (EGF), 260‐mM l‐glutamine, 2% B‐27 supplement without vitamin A (Invitrogen), 250‐µM/ml amphotericin, and 50‐mg/ml gentamycin (Fisher), and incubated in 10% CO_2_ at 37°C. Medium was changed daily for 2 days, then weekly. Gliospheres were maintained in vitro for 5–7 days before intracranial injection. For intracranial injections, cells were dissociated with Accutase (Sigma‐Aldrich), counted, and then diluted in sterile phosphate‐buffered saline (PBS) to get desired cells/unit volume.

For visualization of pediatric tumor cells, PD456 and PD2159 were transduced with lentiviral vectors to facilitate visualization in situ. Concentrated CSCW2‐IRES‐mCherry lentivirus (MGH Vector Core Facility, NIH/NINDS P30NS045776) was combined with 8 µg/µl hexadimethrine bromide (Sigma) in cell culture media and added to the tumor cells for 24 hr. The virus was then removed and fresh cell culture medium was added. Fluorescence Activated Cell Sorting (Sony SH800) was used 5–7 days posttransduction to isolate a mCherry^+^ population for each tumor cell line. The generated lines were subsequently maintained as xenografts, as described above. For immunofluorescence, slices were blocked using 3% bovine serum albumin (BSA) in 1 × tris‐buffered saline (TBS) plus with 0.1% Triton and incubated overnight at 4° with primary antibodies for human‐specific Nuclei (HuNu, 1:250; Cat. MAB1281, Millipore).

### 3.7 Intracranial glioma injections

Human pediatric glioblastoma PD456 and PD2159 tumors, previously established from human biopsies, were implanted into the cerebrum of postnatal day 2–3 and 17–18 old pups, and 6–7‐week‐old immunodeficient C.B.17 *scid* mice. Briefly, adult male and female mice were anesthetized with 2%–5% isoflurane and fixed to a stereotaxic apparatus (Leica Angleone stereotaxic model 39464710) followed by a midline scalp incision under aseptic conditions. Stereotactic coordinates used were as follows: 1.0–2.0 mm lateral and 0.5–1.0 mm anterior to bregma and −1.4 mm deep. Patient‐derived xenograft tumor cells (2.0 × 10^5^ cells in 2 μl of PBS, PD456 or PD2159) were injected at a depth of 2.0–2.5 mm. At the completion of infusion, the syringe needle was allowed to remain in place for 2 min, then slowly withdrawn to minimize backflow off the injected suspension. Xenografts for pups were done using similar techniques with modifications to adjust for the size of the brain. In brief, pups were anesthetized with 2%–5% isoflurane and put on a heating pad with continuous supply of isoflurane. Stereotactic coordinates used were as follows: 1.0–1.2 mm lateral and 0.5–0.8 mm anterior to bregma and 0.5–0.8 mm deep and position was marked by pen without opening the skin. Animal was held by one hand followed by injections with steady hand using a depth stopper syringe (Hamilton™ Neuros™ 700 Series Microliter Syringes #‐ 65460‐02). All control mice were injected with sterile suspension media used for glioma cells. Body weight of animals was measured on alternate days and experiments were conducted between 8 and 9 days postglioma implantation. A 10‐µl syringe (World Precision Instruments #SGE010RNS) was used to infuse glioma cells at 11 nl/s rate.

### 3.8 Acute slice electrophysiology

At 7–8 days after xenografting, coronal brain slices were prepared (p9‐10, p17/18, p35‐40) in accordance with approved protocol. After cervical dislocation, mice were quickly decapitated and brains were dissected and kept in an ice‐cold ACSF (135 mM NMDG, 1.5 mM KCl, 1.5 mM KH_2_PO_4_, 23 mM choline bicarbonate, 25 mM d‐glucose, 0.5 mM CaCl_2_, 3.5 mM MgSO_4_; pH 7.35, 310 ± 5 mOsm; Sigma‐Aldrich) saturated with carbogen (95% O_2_ +5% CO_2_). Coronal slices (300 μm) were prepared using a Leica VT 1000P tissue slicer and slices were allowed to recover for 1 hr in ACSF (125 mM NaCl, 3 mM KCl, 1.25 mM NaH_2_PO_4_, 25 mM NaHCO_3_, 2 mM CaCl_2_, 1.3 mM MgSO_4_, 25 mM glucose, pH 7.35, 310 ± 5 mOsm) at 32°C. Afterwards slices were kept at room temperature until used for recordings. Individual slices were placed in a recording chamber and continuously superfused with ACSF at a flow rate of 2 ml/min. Glioma cells grew rapidly and expanded to primary motor and somatosensory cortical areas after 7 days postinjection. Tumor masses in these cortical areas were visually identified by their unique appearance as described previously (Stone et al., 2018). The pyramidal cells in cortical layers 2/3 were identified under an upright microscope (Leica DMLFSA) with a X40 water immersion lens and infrared illumination. Whole‐cell voltage‐clamp and current‐clamp recordings were achieved using an Axopatch 200B amplifier (Molecular Devices). Patch pipettes of 3–5 MΩ open‐tip resistance were created from standard borosilicate capillaries (WPI, 4IN THINWALL Gl 1.5OD/1.12ID) using Narishige PP‐83 and HEKA PIP 6 vertical pipette pullers. Patch pipettes were filled with an intracellular solution of 134 mM potassium gluconate, 1 mM KCl, 10 mM 4‐(2‐hydroxyethyl)‐1‐piperazineethanesulfonic acid (HEPES), 2 mM adenosine 5′‐triphosphate magnesium salt (Mg‐ATP), 0.2 mM guanosine 5′‐triphosphate sodium salt (Na‐GTP) and 0.5 mM ethylene glycol tetraacetic acid (EGTA; pH 7.4, 290–295 mOsm). Patch pipettes were visually guided using MM‐225 micromanipulator (Sutter Instrument, Navato, CA). Whole‐cell recordings were made once a >5–10 GΩ seal was achieved. For voltage‐clamp recordings, the membrane potential was clamped at −70 mV. The membrane capacitance (Cm) and series resistance were not compensated unless otherwise stated. Data were acquired using Clampex 10.4 software and Axon Digidata 1550A interface (Molecular Devices), filtered at 5 kHz, digitized at 10–20 kHz and analyzed using Clampfit 10.6 software (Molecular Devices). During all the recordings carbogen‐bubbled ACSF was continuously superfused (2 ml/min) and all recordings were made at 32–33°C using an inline feedback heating system (Cat# TC 324B, Warner Instruments).

### 3.9 Data analysis

The resting membrane potential (Vm) was measured by setting *I* = 0 mode immediately after achieving whole cell. Action potential (AP) threshold current was calculated by injecting 2 – 200 pA current pulses (10 ms duration) with 2 pA increments in each step. Minimum current required to generate first AP was denoted as threshold current. Input resistance (*R*_in_), was determined by injecting 15 hyperpolarization current steps (−100 pA each for 1,000 ms) and the steady‐state membrane voltage deflection (Δ*V*) was recorded. The *R*_in_ was measured as a ratio (Δ*V*/*I*) of the steady‐state change in the membrane voltage (Δ*V*) and the corresponding injected current (*I*). Action potential firing properties of neurons were assessed using the input–output curve obtained by applying increasing current steps of different magnitudes (−100 to 240 pA, 20 pA increment, duration 1,000 ms) and counting the number of APs using a Clampfit 10.6 program. We observed that the amplitudes of APs shortened as the firing frequency increased; therefore, we set a minimum 15 mV deflection from the steady‐state response as a qualifying criterion for a spike to be identified as an AP.

### 3.10 Statistical analysis

Data are represented as box and whisker plots unless otherwise stated in the specific figures. Experimental designs with two treatment groups were analyzed by two‐tailed unpaired or paired *t* tests. Welch's correction was applied where variances of both the groups were statistically different. Experimental designs with more than two groups were analyzed using one‐way ANOVA or two‐way ANOVA followed by appropriate post hoc multiple comparison tests. Statistically significant difference between groups were noted in graphs using asterisk(s) (*p < .05, **p < .01, ***p < .001,) and occasionally number sign(s) (^#^ *p* < .05, ^##^ *p* < .01, ^###^ *p* < .001,). Data analysis was performed using GraphPad Prism 7.0, Microsoft Excel, and Origin 2016 (OriginLab) by two individuals who were blinded to the experimental groups. All data are presented as mean ± standard error of mean. *N* values represent the number of neurons/number of animals.

## CONFLICT OF INTEREST

None declared.

## AUTHOR CONTRIBUTION

L.T. performed tumor implantations, electrophysiology recordings, and data analysis. B.P.T. performed data analysis and wrote manuscript. A.B. conducted electrophysiology experiments. A.S. performed data analysis. A.G. conducted electrophysiology recordings. E.G.T. labeled glioma cells with mCherry. N.F. performed IHC experiments. S.L.C. conceived idea, experimental design, data interpretation, and wrote manuscript.
